# Predictors of contraceptive method mix in the Cameroon development corporation plantation camps

**DOI:** 10.11604/pamj.2021.38.156.22357

**Published:** 2021-02-11

**Authors:** Agbor Nathan Emeh, Dickson Shey Nsagha, Hermann Ngouakam

**Affiliations:** 1 Department of Public Health and Hygiene, Faculty of Health Sciences, University of Buea, P.O. Box 12, Buea, Cameroon

**Keywords:** Socio-demographic, determinants, contraceptive method mix, reproductive health

## Abstract

**Introduction:**

low socioeconomic status is a risk factor for maternal death and contraceptive use has been shown to reduce maternal deaths in those poor settings. Despite the tremendous benefits of contraceptives in the regulation of reproductive health indicators, its use in less developed countries continue to remain unacceptably low. The purpose of this study was primarily to assess the contraceptive method mix and then determine the predictors of contraceptive use in the Cameroon Development Corporation (CDC) plantation camps.

**Methods:**

mix sampling was used. Firstly, two CDC camp localities (Tiko and Pena Mboko) were purposively selected. Pre-existing clusters within these localities were then randomly selected and then eligible participants within the sampled clusters systematically selected. Using the main street junction as starting point, direction of sample collection was determined by spinning a plastic bottle. From the start of street junction and moving in direction of the bottle pointer, all households left to the principal investigator were visited in search of eligible participants which were sexually active women aged 15-49 years who gave consent/assent. One participant was selected per household. We used pretested interviewer-administered questionnaires that covered information on socio-demographic characteristics, reproductive health and contraceptive use. Statistical significance was set at p-value ≤ 0.05.

**Results:**

six hundred and thirty four (634) sexually active women aged 15-49 years were included in the study; majority were 25-35 years (246; 38.8%). The current contraceptive prevalence was 63.1% [59.3-66.8] (400); of which 312 participants (78%) used a single method while 88 (22%) participants used contraceptives in combination. The most common methods in use were rhythm (196; 49%), male condom (109; 27.2%) and implants (63; 15.8%). When adjusted, statistically significant determinants for contraceptive use were age range and marital status such that odds of using contraceptives was lower in women < 35 years and those cohabiting (AOR= 0.71 [0.50-1.00] and AOR=0.62 [0.44-0.87] respectively).

**Conclusion:**

current contraceptive practice in the CDC plantation camps is geared toward less effective traditional methods than the more effective modern methods. More health education is needed to adjust this paradigm.

## Introduction

Although family planning has always been a major tool in the sustenance and evaluation of global health, universal access to reproductive health, including voluntary access to family planning as postulated in Millennium Development Goal (MDG) 5B was not achieved by 2015 particularly in Sub-Saharan Africa [1] where its access continues to remain a major challenge. However, in recent years, several different partnership programs have mobilise effort globally to increase the access to reproductive health, including “Global Strategy for Women´s and Children´s Health”, Family Planning 2020 (FP2020), the MDG Health Alliance, the United Nation (UN) Commission on life-saving commodities and others [2]. According to the FP2020 global partnership program, every woman should have access to lifesaving contraceptives, no matter where they live [3]. This goal of the FP2020 complements the reproductive health goal targets postulated in the Sustainable Development Goal (SDG) 3 and 5. In other to achieve this universal family planning access goal, FP2020 program has involved a wide range of partnership including multilateral development agencies, donor governments and private philanthropists, implementing partners, civil society stakeholders [3]. Although goals relating to sexual and reproductive health are SDG 3 (3.1, 3.7 and 3.8) and SDG 5 (5.6) [4], in an analysis, Ellen insisted on the key role of family planning to achieving all the 17 SDGs and their indicators [2]. World Health Organization (WHO) have postulated a set of 17 indicators that covers the main areas of reproductive health of which half can be directly or indirectly impacted by family planning [5]. These indicators include total fertility rate, contraceptive prevalence, maternal mortality ratio, perinatal mortality, Prevalence of low birth weight, Percentage of obstetric and gynecological admissions owing to abortion, Reported incidence of urethritis in men, prevalence of HIV infection in pregnant women.

According to the International Federation of Gynecology and Obstetrics (FIGO), over 885 million women of reproductive age in low income countries currently want to avoid pregnancy yet their unmet need for modern contraception stands to remain very high [6]. Globally, over 10% of all women do not have access to an effective method of contraception [1]. Unplanned pregnancy is a major cause of maternal morbidity and mortality [7]. When pregnancies are planned and spaced using modern contraceptives, the risk of maternal and neonatal mortality drops [6] and the woman and her children tend to experience better health and social outcomes [6]. Maternal mortality is estimated to be about 1.8 times higher in women not using modern contraceptives than among modern contraceptive users [8].

Maternal mortality in Cameroon continue to remains unacceptable high [9] yet the prevalence of modern contraception has shown only slow increase [10]. Despite the availability of a wide range of contraceptive methods, contraceptive use is very low [11,12] and the rate of unintended pregnancies continue to remain high, particularly in developing countries [7]. The main contraceptive methods in use in Cameroon include implants, intra-uterine device (IUD), combined oral contraceptives (COC), hormonal injectable methods and condoms [13]. There appear to be an increasing tendency in the use of modern contraception over time and a decrease in the use of tradition methods in Cameroon. While the general contraceptive use in Cameroon has increased from 16.1% in 1991 to 23.4% in 2011, the use of modern methods has increased from 4.3% in 1991 to 14.4% in 2011 and traditional methods decreased from 11.8% in 1991 to 8.9% in 2011 [10]. Main reasons for non-use of contraceptives by those in need of contraception include health concerns, infrequent sex, opposition from others, lack of access or knowledge [11]. Several factors have been associated with the use of contraceptives such as age of woman, marital status, partner´s approval of contraception and discussion of family planning with couple [14]. Other reasons for use of a particular contraceptive method in Cameroon include the effectiveness of the method, its financial and geographical accessibility, its ability to prevent sexually transmitted infections and whether or not it is prescribed by a medical personnel [15].

Although there is an increasing trend of studies addressing contraception worldwide, literature on this subject matter is scanty in Cameroon. There are no studies on contraception in cohort lifestyle in Cameroon and the prevalence of contraceptive use, the nature and the determinants of contraception in Cameroon Development Corporation (CDC) is unknown. The purpose of this study is to assess the contraceptive method mix and socio-demographic determinants associated with contraceptive use in CDC plantation camps.

## Methods

**Study area:** Tiko which has a total surface area of 4,840 km^2^ is bounded to the West by Limbe, to the North by Buea, to the North-East by Muyuka, to the East by Dibombari in Mungo Division and to the South by Bonaberi. Likewise, Penda Mboko is situated in the Mungo Division of the Littoral region of Cameroon with geographical coordinates 40 16' 35" North, 90 26' 49" East. Within these localities are CDC plantation camps that provide settlements for thousands of labourers. The main produce of CDC are rubber, banana and palm oil.

**Figure 1:**
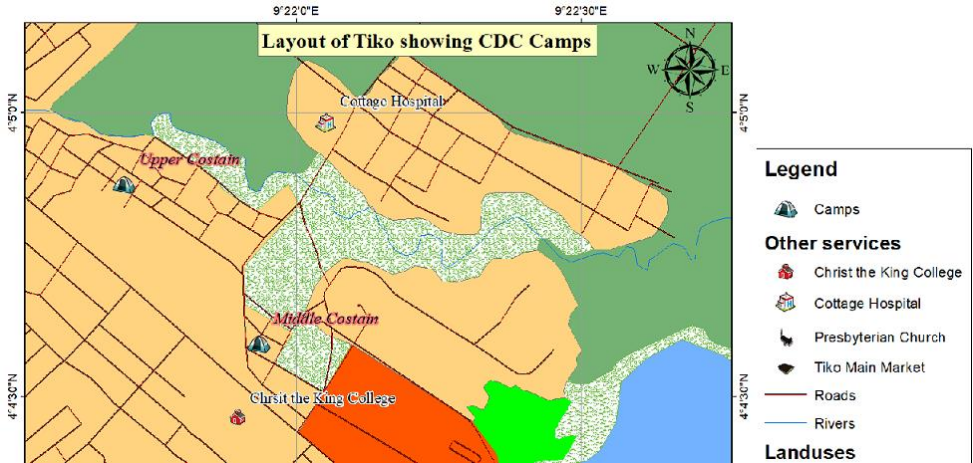
layout of Tiko CDC camps

**Figure 2:**
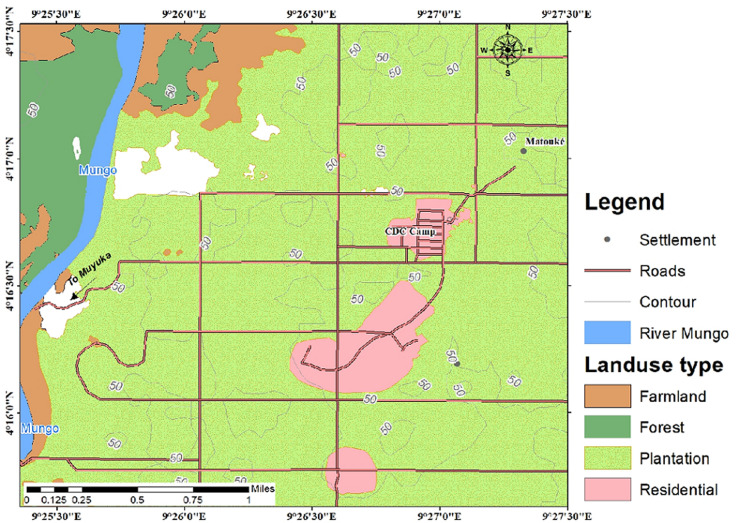
Pena Mboko CDC plantation camps

**Study design and setting:** this was a community-based cross sectional study in which participants were enrolled from CDC setting areas of Tiko and Pena Mboko (Figure 1 and Figure 2). The participants were interviewed in their households between December 2019 and February 2020.

**Sample size determination:** the minimum sample size for the study was calculated based on the following statistical formula [16]:

$$n=\frac{Z^{2}P(1-P)(Deff)}{d^{2}}$$

Where Z= 1.96 at 95% confidence interval, P=23%= 0.23 (prevalence of contraceptive use in Cameroon) [10], d=5%=0.05 (error margin), deff (design effect) =1.1.

$$n=\frac{{\left(1.96\right)}^{2}(0.23)(0.77)(1.1)}{{\left(0.05\right)}^{2}}=300\text{ participants}$$

However, a total sample of 634 women of reproductive age in both Tiko and Pena Mboko CDC camps were included in the study.

**Sampling technique:** two CDC localities (Tiko and Pena Mboko) located in two different regions (South West and Littoral Region respectively) were purposively selected since these are the main camps existing in these two regions. Each of these camps was divided into clusters which represented their pre-existing camp quarters. Tiko has nine (9) clusters from which four (4) were randomly selected using the simple balloting method. Likewise, Pena Mboko has seven (7) clusters from which four (4) were also randomly selected using the same method. Within the selected clusters, the main road junction was identified and then by spinning a plastic bottle at the identified junction, using the bottle head as the direction pointer, a direction was also chosen. By moving in the selected direction, beginning from the start of the junction, all households at the left hand side of the principal surveyor were systematically visited. At the level of the household, only a single eligible participant per household was selected for interview and this was the first eligible participant encountered in the household by the surveyor. The number of selected participants per cluster was proportionate to the estimated population size of the cluster.

**Data collection procedures:** after collecting the ethical clearance and administrative authorizations, community chiefs and quarter heads were visited from whom authorization to access the communities was obtained. Access into the communities were led and guided by community health workers (CHWs) of the Tiko and Pena Mboko health districts in their respectively districts. Trained data collectors collected data in the two districts simultaneously throughout the study period. Surveyors visited each sampled households and inquired whether or not there was a potential participant (women of reproductive age) in the household. In households were participants were presently on sit, data was collected otherwise, surveyors visited the sampled household as many times as possible until eligible participant was contacted. Only one participant was selected per household, and this was the first eligible participant encountered. In cases where the first contacted eligible participant in a particular household refused to participate in the study, subsequent eligible participants of the same household were contacted. After providing consent/assent to participate in the study, they were required to provide information for the study. Data were collected using pre-tested interviewer administered semi-structured questionnaire. The questionnaire covered socio-demographic information of the study participants including age, marital status, education and income level. The questionnaire also covered reproductive health indicators and history of contraception and the contraceptive methods used.

**Target population:** those included for the study were sexually active women aged 15-49 years, resident within the Tiko and the Pena Mboko CDC camps and who provided consent/assent to be included in the study.

**Operational terms:** contraception: contraception is a way to prevent pregnancy [17]. This is achieved by the use of contraceptive methods of which there exist over 15 different methods from which a woman can make her choice [18]. Contraceptive method mix: the per-cent distribution of contraceptive users (or alternatively, of first-time users) by method in a defined period [19]. Prevalence = (number of users of a specific method/total number of contraceptive user) x 100. Reproductive age Group: reproductive age group is often used to refer to women who are in the age range of 15-49 years [5]. This ideally represents the age at which a woman is reproductive, although cases of reproduction have been reported in women less than 15 years and greater than 49 years. Married: a woman of reproductive age who is in legal relationship with a male partner. Co-habiting: refers to a woman of reproductive age who is in a marriage-like relationship with a male partner and is not married to or in a registered partnership with each other. Sexually active: a woman who had her first sexual contact at least three months before onset of the study [20].

**Ethical considerations:** ethical approval for the study was obtained from the Institutional Ethics Committee for Research on Human Health of the University of Douala (Ref. no: 2069_IEC-UD/12/2019/T). Administrative authorizations were obtained from the regional delegation of Health of the South West Region, Littoral Region and the Director of Human Resources of the CDC. Only consenting women were included in the study and for those who were aged below 18 years, assent was obtained from their parents or legal representatives.

**Data management and statistical analysis:** all the questionnaires were manually checked by the principal investigator before they were being introduced into the statistical software. Those questionnaires that lacked vital information such as participant age, code number, etc. were not analysed. Data were entered into and cleaned in EpiData 3.1, then exported and analysed in SPSS version 16. Summary result was presented in tables and statistical significance was considered at p<0.05. Categorical variables were described using frequencies and their 95% confidence intervals while continuous variables were described using mean. Regression analysis was used to determine the predictors (independent variables) of contraceptive use (outcome variable); in the univariate analyses, odd ratio (OR) and 95% confidence interval were used to assess the relationship between predictors and contraceptive use. Adjusted OR was calculated by simultaneously introducing confounders (covariates for which the univariate analyses showed statistically significant association with contraceptive use) into the multiple logistic regression model.

## Results

**Demographic characteristics of study participants:** during the study, six hundred and thirty nine (639) women aged 15-49 were interviewed. However, we excluded 5 questionnaires during data analysis due to incompleteness of information, leaving 634 participants as our final sample (Table 1). The mean age of our study participants was 31.42±8.3, majority [246: 38.8%] were those aged 25-35 years. Three hundred and thirty four [334: 52.7%] participants were married women, 79 (12.46%) were co-habiting and 184 (29%) single. Majority of the participants [265: 41.8%] had primary level of education while 173 (27.3%) had none. Most of the participants were pentecostals [425: 67%] and Catholics [186: 29.3%]. While majority of the participants [282: 44.5%] had a monthly income ranging from 25000-50000FCFA, only a few [54: 8.5%] could earn more than 75000FCFA a month.

**Table 1:** socio-demographic characteristics of women aged 15-49 in Tiko and CDC plantation camps

Variables	Tiko №(%)		Pena Mboko №(%)	Total №(%)	p-value
Age Range (years)					
< 25	103(30.9)		77(25.6)	180(28.4)	0.001
25-35	107(32.1)		139(46.2)	246(38.8)	
>35	123(36.9)		85(28.2)	208(32.8)	
Total	333 (100)		301(100)	634(100)	
Marital Status					
Married	167(50.2)		168(55.8)	335(52.8)	0.294
Co-habiting	42(12.6)		37(12.3)	79(12.5)	
Single	107(32.1)		77(25.6)	184(29)	
Divorced/Widowed	17(5.1)		19(6.3)	36(5.7)	
Total	333(100)		301(100)	634(100)	
Education Level					
None	77(23.1)		96(31.9)	173(27.3)	0.035
Primary	142(42.6)		123(40.9)	265(41.8)	
Secondary	62(18.6)		52(17.3)	114(18)	
Tertiary	52(15.6)		30(10)	82(12.9)	
Total	333(100)		301(100)	634(100)	
Religion					
Catholic	81(24.3)		105(34.9)	186(29.3)	0.004
Pentecostals	236(70.9)		189(62.8)	425(67)	
Muslim	7(2.1)		6(2)	13(2.1)	
None	9(2.7)		1(0.3)	10(1.6)	
Total	333(100)		301(100)	634(100)	
Income Level (FCFA)					
<25000	70(21)		70(23.3)	140(22.1)	0.511
25000-50000	153(45.9)		129(42.9)	282(44.5)	
50000-75000	78(23.4)		80(26.6)	158(24.9)	
>75000	32(9.6)		22(7.3)	54(8.5)	
Total	333(100)		301(100)	634(100)	
					

**Figure 3:**
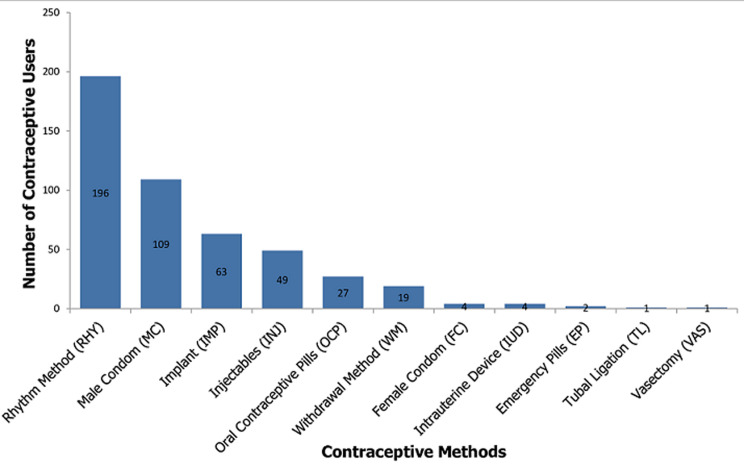
a chart of frequency of contraceptive method mix in the CDC plantation camp

**Contraceptive method mix of women aged 15-49 years in CDC plantation camps:** of the 634 participants, four hundred participants [400; 63.1% (59.3-66.8)] were currently using at least one contraceptive method at the time the study. The contraceptive prevalence was slightly higher in Pena Mboko [198/301; 65.8% (60.3-70.9)] than in Tiko CDC camps [202/333; 60.7% (55.3-65.8)] though this was not statistically significant (p=0.09). Among the 400 contraceptive users, 312 (78%) used a single method of contraception while 88 (22%) participants used contraceptives in combination (Table 2 and Figure 3). Table 2 shows the different combinations of contraceptives used by participants. Contraceptive use was highest among women aged 25-35 years [167/246; 67.9%] followed by those < 25 years [111/180; 61.7%] and lowest among the older women of reproductive age [122/208; 58.7%] although this was not statistically significant (p=0.48).

**Table 2:** contraceptive method mix among contraceptive users in CDC plantation camps

Contraceptive methods: n(%)	MC	FC	OCP	INJ	IUD	IMP	WM	RHY	EP	TL	VAS	Total (n=400)
Male condom (MC)	46(11.5)	1(0.3)	1(0.3)	1(0.3)	0(0.0)	2(0.5)	9(2.3)	47 (11.8)	1(0.3)	0(0.0)	1(0.3)	109 (27.2)
Female Condom (FC)	1(0.3)	2(0.5)	0(0.0)	0(0.0)	0(0.0)	0(0.0)	0(0.0)	1(0.3)	0(0.0)	0(0.0)	0(0.0)	4 (1.0)
OCP	1(0.3)	0(0.0)	21(5.3)	0(0.0)	0(0.0)	0(0.0)	2(0.5)	3(0.8)	0(0.0)	0(0.0)	0(0.0)	27(6.8)
Injectables (INJ)	1(0.3)	0(0.0)	0(0.0)	44(11.0)	0(0.0)	0(0.0)	0(0.0)	4(1.0)	0(0.0)	0(0.0)	0(0.0)	49(12.2)
IUD	0(0)	0(0.0)	0(0.0)	0(0.0)	4(1.0)	0(0.0)	0(0.0)	0(0.0)	0(0.0)	0(0.0)	0(0.0)	4(1.0)
Implant (IMP)	2(0.5)	0(0.0)	0(0.0)	0(0.0)	0(0.0)	61(15.3)	0(0.0)	0(0.0)	0(0.0)	0(0.0)	0(0.0)	63(15.8)
WM	9(2.3)	0(0.0)	2(0.5)	0(0.0)	0(0.0)	0(0.0)	0(0.0)	8(2.0)	0(0.0)	0(0.0)	0(0.0)	19(4.8)
Rhythm method (RHY)	47(11.8)	1(0.3)	3(0.8)	4(1.0)	0(0.0)	0(0.0)	8(2.0)	132 (33.0)	1(0.3)	0(0.0)	0(0.0)	196(49)
Emergency pills (EP)	1(0.3)	0(0.0)	0(0.0)	0(0.0)	0(0.0)	0(0.0)	0(0.0)	1(0.3)	0(0.0)	0(0.0)	0(0.0)	2(0.5)
Tubal ligation (TL)	0(0.0)	0(0.0)	0(0.0)	0(0.0)	0(0.0)	0(0.0)	0(0.0)	0(0.0)	0(0.0)	1(0.3)	0(0.0)	1(0.3)
Vasectomy (VAS)	1(0.3)	0(0.0)	0(0.0)	0(0.0)	0(0.0)	0(0.0)	0(0.0)	0(0.0)	0(0.0)	0(0.0)	0(0.0)	1(0.3)
Total (n=400)	109(27.2)	4(1.0)	27(6.8)	49(12.2)	4(1.0)	63(15.8)	19(4.8)	196(49)	2(0.5)	1(0.3)	1(0.3)	475 (100)

OCP=oral contraceptive pills; IUD= intrauterine device; WM= withdrawal method

Among the 400 contraceptive users, the most prevalent methods in use were the rhythm method [196; 49%], male condom [109; 27.2%], implants [63; 15.8%] and injectable contraceptive [49; 12.2%]. See Table 2. The sum total of the contraceptive methods in use (475) is more than the number of contraceptive users (400). This is because some user used more than a single method of contraceptive at the time of study for one reason or another. The percentages in Table 2 represent the proportion of contraceptive users who utilise a particular contraceptive method. We noticed a decreasing trends in prevalence of male condom use with age such that its use was more among those less than 25 years ( 43.2%), followed by those 25-35 years (25.7%) and then those greater than 35 years (14.8%) (P-value=0.00). On the contrary, there was an increasing trend in the prevalence of implants use with age such that 13.5% of those less than 25 years used implants, 14.4% of those 25-35 years and then those greater than 35 years (19.7%) (P-value=0.19). There was a decreasing trend in the proportion of male condoms users with increasing income levels up to a monthly income of 75000FCFA and then increased for those with income levels more than 75000FCFA as follows: 29.9%, 28.4%, 21.3% and 34.5% for income level 'less than' 25000FCFA, 25000-5000FCFA, 50000-75000FCFA and 'greater than' 75000FCFA respectively (p-value=0.56). The highest proportion of male condom users were those who were single [46/104; 44.2%] followed by those who were co-habiting [17/55; 30.9%] (p-value of 0.00). Among those who were married, 20.2% [45/223] used male condom at the time of the study. Condom use was least among the widows (5.6%).

**Predictors of contraceptive use of women aged 15-49 years:** Table 3 presents the predictors of contraceptive use in the CDC plantation camps. Among the different predictors assessed, statistically significant determinants of contraceptive use were living with a partner (OR; 1.64[1.17-2.30], p=0.00), getting into union at an age less than 30 years (OR; 2.26 [1.06-4.78], p=0.03) and having last pregnancy not more than 5 years ago (OR; 1.48[1.03-2.15], p=0.04). However, after adjusting for confounders, statistically significant predictors were participants with age less than 35 years (AOR; 0.71 [0.50-1.00], p=0.04) and living with a partner (AOR; 0.62 [0.44-0.87], P=0.01).

**Table 3:** socio-demographic and reproductive health determinants of general contraceptive use among women aged 15-49 years in the CDC plantation camps

Variables	Unadjusted OR [95%CI]	p-value	Adjusted OR [95%CI]	p-value
Participant having age < 35 years (Y/N)	1.32[0.94-1.86]	0.11	0.71[0.50-1.00]	0.04*
Living with a partner (Y/N)	1.64[1.17-2.30]	0.00*	0.62[0.44-0.87]	0.01*
Level of education below primary (Y/N)	1.20[0.85-1.69]	0.31	0.91[0.64-1.30]	0.61
Pentecostals (Y/N)	0.75[0.53-1.07]	0.11	1.35[0.95-1.92]	0.09
Having income level < 50000FCFA (Y/N)	0.91[0.64-1.28]	0.57	1.10[0.78-1.55]	0.59
Living in Tiko CDC Camp (Y/N)	0.80[0.58-1.11]	0.18	1.22[0.88-1.69]	0.24
Age of getting into union< 30 years (Y/N)	2.26[1.06-4.78]	0.03*	0.50[0.23-1.07]	0.07
First Sexual Contact age < 20 years (Y/N)	1.13[0.57-2.26]	0.73	0.98[0.46-2.09]	0.95
Nulli/primiparous (Y/N)	0.76[0.54-1.08]	0.12	1.21[0.76-1.94]	0.42
Having < 3 Children (Y/N)	0.71[0.47-1.07]	0.10	1.32[0.87-2.02]	0.20
Last pregnancy outcome: Singleton (Y/N)	1.53[0.90-2.61]	0.12	0.62[0.36-1.07]	0.09
Last pregnancy :< 5 years ago (Y/N)	1.48[1.03-2.15]	0.04*	0.70[0.48-1.02]	0.06
Number of abortions: < 3 Abortions (Y/N)	2.61[0.73-9.33]	0.14	0.39[0.11-1.40]	0.15
Intend Getting Pregnant at this time (Y/N)	0.96[0.35-2.68]	0.94	0.96[0.33-2.83]	0.94

* = Statistically significant predictors

## Discussion

There has been an increasing trend of studies with interest in contraception globally motivated by the notion of contraceptive being “best buy” for the achievement of the Sustainable Development Goals (SDGs) [2]. However, sufficient interest has not been place on this subject in the less develop countries, especially in Cameroon where only few studies on this are available in search engines [14,15,20,21]. This paper addresses a six (6) months contraceptive method mix in the Tiko and Pena Mboko CDC plantation camps and the socio-demographic determinants for general contraceptive use by resident reproductive age group women in the CDC plantation camps.

This study showed that at the time of data collection 63.1% were currently using at least one contraceptive method. An earlier report had shown increasing trend of general contraceptive prevalence in Cameroon from 16.1% in 1991 to 23.4% in 2011, with increasing trends more in the urban than in the rural settings [10]. Most recent studies that have evaluated the use of contraception have reported contrasting prevalence in different areas in Cameroon, with some being as low as 18.3% [13] and some as high as 69.6% for general contraceptive use [14]. These differences in prevalence of contraceptive could be accounted for by the differences in locality and the time when the study was carried. Studies has shown that contraceptive use is often higher in urban than in rural settings and also that the general contraceptive use is increasing over the years [10,22] such that the contraceptive prevalence in more recent studies are generally higher than in earlier studies. However, this is not a general rule since the current contraceptive prevalence in certain developing countries continues to remain particularly low. The contraceptive methods most in used at the time of this study were rhythm method, male condom, implant, injectable and oral contraceptive pills with rates of 49.0%, 27.2%, 15.8%, 12.2% and 6.8% respectively. The contraceptive methods in use in a particularly locality vary greatly base on several factors, notably the availability of contraceptive methods and whether or not the health personnel are experienced in the methods available [15,20], such as in the case of implants and intrauterine devices where low experience of health personnel in placing these methods often make their prevalence particularly low in most community based studies. Other forms of contraceptives including contraceptive patch, cervical ring/cap, diaphragm, contraceptive sponge, and contraceptive film were not used by the participants in the camps. However, some participants used certain substances to prevent them from getting pregnant when they felt they were at risk of pregnancy. These substances have never been reported in literature to be having contraceptive properties. They included high doses of paracetamol [2/400, 0.5%], high concentration of salt and water [1/400, 0.25%]. In a study carried out in an urban setting in Cameroon [15], the most common reversible contraceptives in use were male condom (76.0%), fertility period based methods (10.1%), female condoms (7.6%), oral contraceptive pills (5.0%), coitus interruptus (4.8%) and implants (4.6%). Other factors may influence the use of a particular contraceptive method in a locality including whether it is being prescribed by personnel in the locality and the price [15].

Several studies have analysed different predictors of contraceptive method use [12,14,20,23]. Predictors of contraceptive use in the CDC plantation camps assessed in this study included age, marital status, education, religion, income level, locality, age at which participant got into union, age of first sexual contact, parity, number of children, outcome of last pregnancy, time when participant had her last pregnancy, number of abortion/miscarriages and whether participants intend to get pregnant anytime soon (Table 3). Univariate analysis showed that marital status and age at which participant got into union were two statistically significant predictors of contraceptive use; those living in union (whether married or consensual union) were 1.6x more likely to use contraceptives than those who were not (p-value=0.00); those who got into union before 30 years of age were 2.3x more likely to use contraceptives than those who got into union after 30 years of age. However, when adjusted, age of participant at the time of study and marital status were the two statistically significant predictor of contraceptive use such that contraceptive use was less likely among those less than 35 years than those greater than 35 years (AOR= 0.71[0.50-1.00], p= 0.004); living in union was less likely to be associated with contraceptive use (AOR= 0.62 [0.44-0.87], p= 0.00). Similar finding was reported an earlier study [14] were women in union were less likely to use contraceptives. Women in union have some form of security in that if they conceive and bear children, their partners may provide some support to the children. This may be the reason why women in union find it unnecessary to prevent pregnancy by using contraceptives. It is well established that maternal age is a risk factor for adverse pregnancy outcomes [24]. The use of contraceptives by women in their later ages will reduce adverse pregnancy outcomes to the same extend at which it reduces high risk pregnancies [25]. Higher use of contraceptive by women of older ages will not be surprising, particularly in settings were women are aware of the higher risk associated with pregnancy at an older age as in the case of CDC plantation camp residents. Although not statistically significant, univariate analysis showed a strong association between contraceptive use and number of abortions/miscarriages such that women who had underwent less than 3 abortions were 2.6X more likely to use contraceptive than those with more than 3 abortions. However, from the study design, it is difficult to determine the direction of the association whether it is the use of contraceptives that is associated with lesser abortions or vis-versa. Literature has shown that rising contraceptive use results in a reduction in the abortion incidence in settings were fertility itself is constant [26,27].

Most studies in Cameroon and Africa in general analysing contraceptive method mix have reported contraceptive users making use of a single contraceptive method at the time of study [13,14,15]. However, at the time of this study, it was noticed that a reasonable proportion of participants made use of more than one contraceptive method. It is a well-accepted fact that no contraceptive method is 100% effective in preventing pregnancy and sexually transmitted infections (STIs). When relying on birth control pills for pregnancy protection, they should be used in combination with a backup contraception, like condom to prevent against sexually transmitted diseases [28,29]. Contraception experts have advised that methods like condom, rhythm method, withdrawal (pullout) method, and other barrier methods should not be used alone [28]. During this study, male condom and rhythm methods were most often used in combination. While only 11.5% of contraceptive users used male condom as their only contraceptive method, 33% of users used rhythm method as their only method of contraception (Table 2). All those who used vasectomy, tubal ligation or IUD to prevent pregnancy did not use them in combination with other contraceptives. This could be a reflection of their perception on the effectiveness of these different methods in preventing pregnancy. Interestingly, as effective as these methods maybe in preventing pregnancies individually, they do not have the ability to protect against STIs and therefore do not exclude them from being used in combination with other barrier methods that could prevent STI like condoms. Literature have cited the use of multiple methods of contraception at a time, most often condom combined with another method [30]. The practice of combined methods in order to increase one´s level of protection from pregnancy and STI is prevalent in some places [29].

## Conclusion

This study assessed the contraceptive method mix and the predictors of contraception use among reproductive age women in the Tiko and the Pena Mboko CDC plantation camps. The overall contraceptive prevalence was 63.1%, with 22% of contraceptive user using contraceptive methods in combination. These combinations may reflect the user´s perception on the effectiveness of the different methods they used in preventing pregnancy and STI depending on their objective, and further studies are needed to proof this. The most common contraceptive method in use was rhythm method, which is a traditional method. Of the modern methods used, male condoms, implants and injectable contraceptives accounted significantly. The major predictors of contraceptive use included age of participant, marital status and the age at which the participant got into union.

### What is known about this topic

- Knowledge and practice of contraceptive in Cameroon, just like in most developing countries is low;
- There is increasing prevalence of contraceptive use in Cameroon;
- Factors determining contraceptive method mix in Cameroon varies from one place to another and this will influence the types of contraceptives being used in that community.

### What this study adds

- Contraceptive prevalence in the CDC plantation camps stood at 63.1% with over 78% of current users using a single method to prevent pregnancy and 22% using contraceptive in combinations;
- Most used methods of contraception in the CDC plantation camps were rhythm method, male condom, implants and injectable;
- A CDC camp resident who was either less than 35 years of age or living with a partner was less likely to use contraceptive.
